# Identification of candidate biomarker MRPL23 and its prognostic potential in non-small cell lung cancer with emphasis on the squamous cell carcinoma subtype

**DOI:** 10.3389/fonc.2026.1663235

**Published:** 2026-02-02

**Authors:** Edyta Podemska, Karol Gostomczyk, Dominika Jerka, Jędrzej Borowczak, Maciej Gagat, Dariusz Grzanka, Justyna Durślewicz

**Affiliations:** 1 Department of Clinical Pathomorphology, Faculty of Medicine, Collegium Medicum in Bydgoszcz, Nicolaus Copernicus University in Torun, Bydgoszcz, Poland; 2 Department of Histology and Embryology, Faculty of Medicine, Collegium Medicum in Bydgoszcz, Nicolaus Copernicus University in Torun, Bydgoszcz, Poland; 3 Department of Tumor Pathology and Pathomorphology, Prof. Franciszek Łukaszczyk Oncology Centre, Bydgoszcz, Poland; 4 Faculty of Medicine, Bydgoszcz University of Science and Technology, Bydgoszcz, Poland; 5 Department of Animal Biotechnology and Genetics, Faculty of Animal Breeding and Biology, Bydgoszcz University of Science and Technology, Bydgoszcz, Poland

**Keywords:** Mrpl23, non-small cell lung cancer, prognostic biomarker, survival & prognosis, TCGA database

## Abstract

**Introduction:**

Lung cancer remains the leading cause of cancer-related mortality worldwide, with non-small cell lung cancer (NSCLC) accounting for the majority of cases. Despite advances in therapy, survival remains poor due to late diagnosis and treatment resistance. Identification of reliable prognostic biomarkers is essential to improve risk stratification and clinical outcomes.

**Methods:**

MRPL23 expression was evaluated as a potential prognostic biomarker in NSCLC using immunohistochemical analysis of tumor specimens from 110 patients and mRNA expression data from The Cancer Genome Atlas (TCGA) cohort. Associations between MRPL23 expression and clinicopathological features, as well as overall survival, were analyzed using survival statistics.

**Results:**

MRPL23 expression was significantly higher in NSCLC tissues than in normal lung tissues (p < 0.0001), with particularly elevated levels observed in squamous cell carcinoma. High MRPL23 protein expression was detected in 57 of 110 cases (51.8%) and was associated with shorter overall survival (median OS 34 vs. 48 months; HR 1.62, 95% CI 1.01–2.58, p = 0.04). These findings were validated in the TCGA cohort, where high MRPL23 mRNA expression correlated with worse overall survival (HR 1.46, 95% CI 1.17–1.83, p < 0.01).

**Discussion:**

MRPL23 overexpression, particularly in lung squamous cell carcinoma, is associated with poor prognosis and may serve as an independent prognostic factor in NSCLC. These results suggest that MRPL23 represents a promising biomarker for improving risk stratification and guiding personalized therapeutic strategies in patients with NSCLC.

## Introduction

Lung cancer, being the foremost cause of cancer-related deaths worldwide, poses a significant public health challenge. The World Health Organization (WHO) reported in 2022 an estimated 2.5 million new cases and 1.8 million deaths attributed to lung cancer globally​ (1)​. This alarming mortality associated with lung cancer can be attributed to several factors. Firstly, frequent disease recurrences and resistance to conventional chemotherapy regimens pose significant obstacles in effectively managing this condition. Furthermore, late-stage detection is a prevalent issue, often leading to limited treatment options and poorer prognoses for patients (2)​. These data elicit significant concern, particularly in light of the substantial advancements in oncology, and strongly underscores the imperative for deeper understanding of the pathobiology of this cancer.

Lung cancer can be divided into two main histological types: small cell lung cancer (SCLCS) and non-small-cell lung cancer (NSCLC). NSCLC represents the predominant subtype, encompassing approximately 80% to 85% of all lung cancer cases, with the remaining 15% comprising SCLC. Within NSCLC, several subtypes can be distinguished, such as adenocarcinoma (LUAD), squamous cell carcinoma (LUSC), and large cell carcinoma (LCLC) (3)​. Typically, in case of early-stage NSCLC, surgical resection is the preferred treatment method, while in advanced-stage lung cancer, the cornerstone of treatment typically involves systemic therapies such as chemotherapy, radiation therapy, or a combination thereof (4). ​Nevertheless, caring about patient’s well-being, therapeutic strategies are individualized, taking into consideration not only tumor stage, histological characteristics, tumor size or location, but also patient-specific attributes such as age, overall health status, and pulmonary function (5). Unfortunately, despite the individualization of therapy and advances in oncological treatment in recent years, the five-year relative survival rate for individuals at all stages of NSCLC is modest 28% (2). ​Therefore, the identification of new prognostic biomarkers is crucial for improving survival and patient care.

The literature highlights certain mitochondrial ribosomal proteins (MRPs) as promising prognostic factors in various cancer types (6–8). Despite advancements in understanding their roles, the functional mechanisms of MRPs are not yet fully elucidated. MRPs play a fundamental role in mitochondrial translation, energy production, and cellular metabolism, underscoring their importance in maintaining cellular homeostasis and functionality. Emerging data also indicate their involvement in the regulation of apoptosis and proliferation (9). This protein family is classified into two subcategories: MRPL (proteins associated with the large ribosomal subunit), and MRPS (proteins associated with the small ribosomal subunit). To date, approximately 80 genes encoding MRPs have been identified, representing key components of the mitochondrial ribosome (10, 11). Among these, MRPL23 has attracted attention as an understudied, yet promising candidate for early cancer diagnostics. Although its role in lung cancer is not well defined, our preliminary TCGA analysis identifies MRPL23 as a potential target, emphasizing its relevance for further research.

Emerging evidence suggests that MRP signaling may contribute to carcinogenesis through multiple interconnected mechanisms (10–12). Since MRPSs, including MRPL23, play a crucial role in the translation of mitochondrial DNA–encoded proteins that are integral to the formation and function of oxidative phosphorylation (OXPHOS) complexes their disruption can alter mitochondrial bioenergetics and initiate a shift toward anaerobic glycolysis (Warburg effect), facilitating disease progression (10, 13, 14). Furthermore, mitochondrial dysfunction associated with aberrant MRPL23 expression may alter reactive oxygen species (ROS) levels, modulating signaling pathways that control apoptosis, proliferation, and genomic stability (15, 16). Elevated MRPL23 expression, as reported in certain cancers, could therefore enhance tumor cell survival by promoting energy production, reducing susceptibility to apoptosis, and fostering a microenvironment conducive to tumor growth (12).

This study was designed to assess the expression of MRPL23 and its potential role as a prognostic biomarker in NSCLC. The primary aim was to determine whether MRPL23 expression correlates with key clinical and pathological features, including overall survival. By examining these associations, we sought to establish whether high MRPL23 expression is linked to worse patient outcomes, supporting its utility as a prognostic marker in NSCLC.

## Results

### MRPL23 protein expression and its association with clinical features and patient survival in the TMA cohort

**Figure 1:**
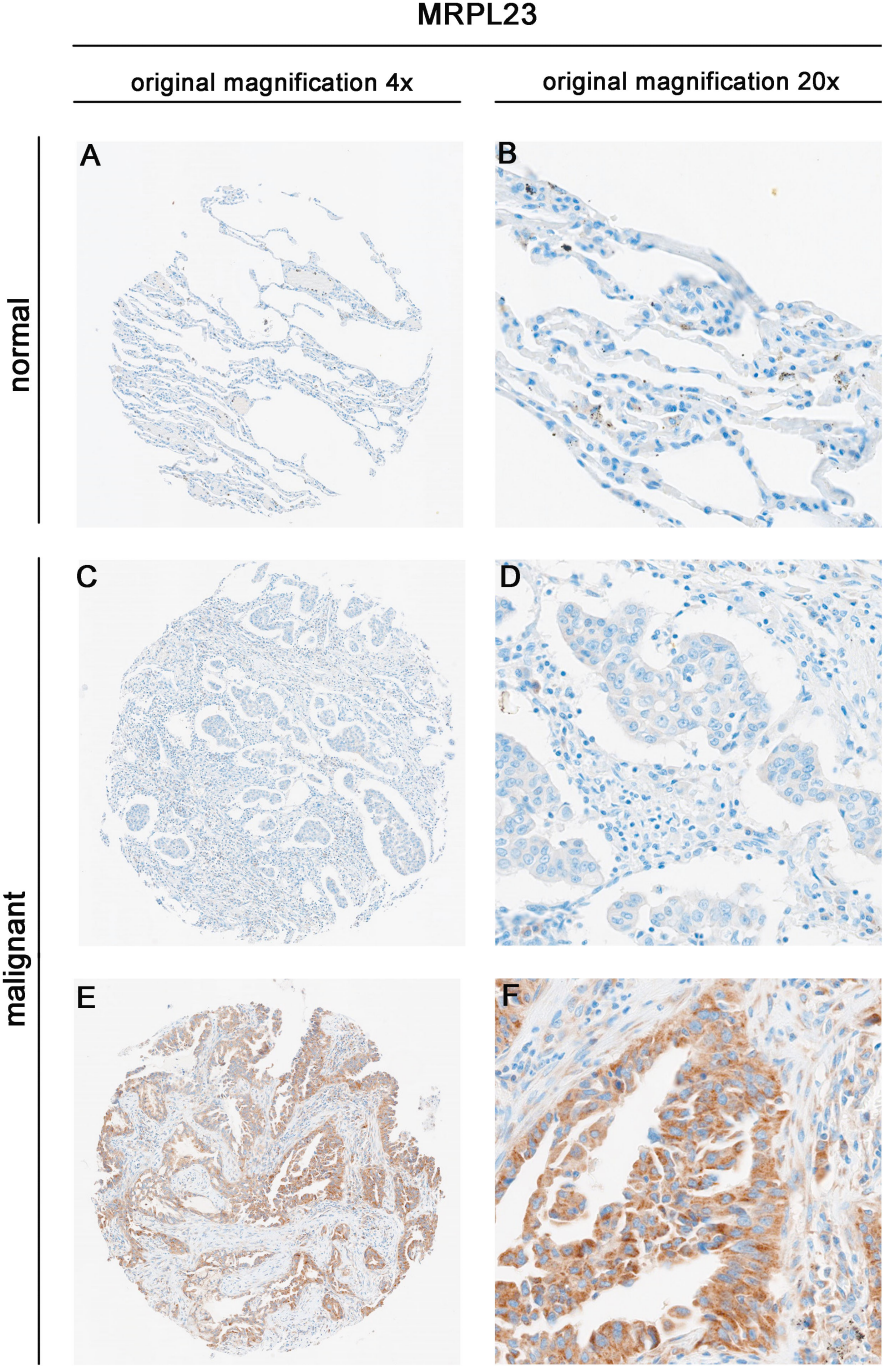
Immunohistochemical expression of MRPL23 in normal lung tissue and non-small cell lung cancer (NSCLC) tissues. Representative images of MRPL23 immunohistochemical staining in normal tissue **(A, B)** and non-small cell lung cancer tissue **(C–F)**. **(C, D)** show low MRPL23 expression, whereas panels E and F show high MRPL23 expression.

**Figure 2:**
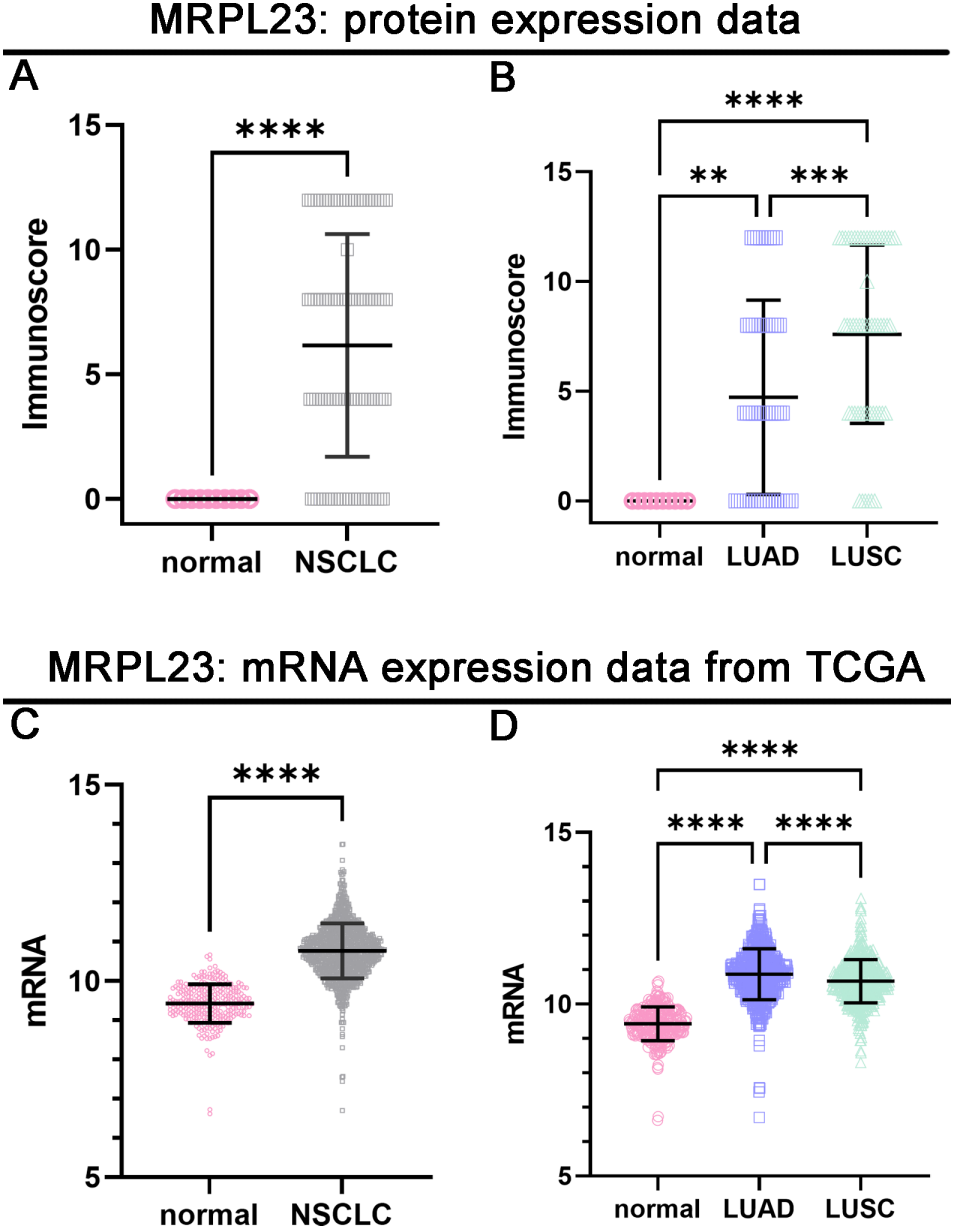
Protein and mRNA expression levels of MRPL23 in normal lung tissue and non-small cell lung cancer (NSCLC) tissues. Protein expression of MRPL23 in NSCLC compared to normal tissues **(A)**. MRPL23 protein expression across normal lung tissue, LUAD (lung adenocarcinoma), and LUSC (lung squamous cell carcinoma) **(B)**. mRNA expression of MRPL23 in NSCLC compared to normal tissues **(C)**. MRPL23 mRNA expression across normal lung tissue, LUAD, and LUSC **(D)**. **p < 0.01, *p < 0.001, ****p < 0.0001.

**Figure 3:**
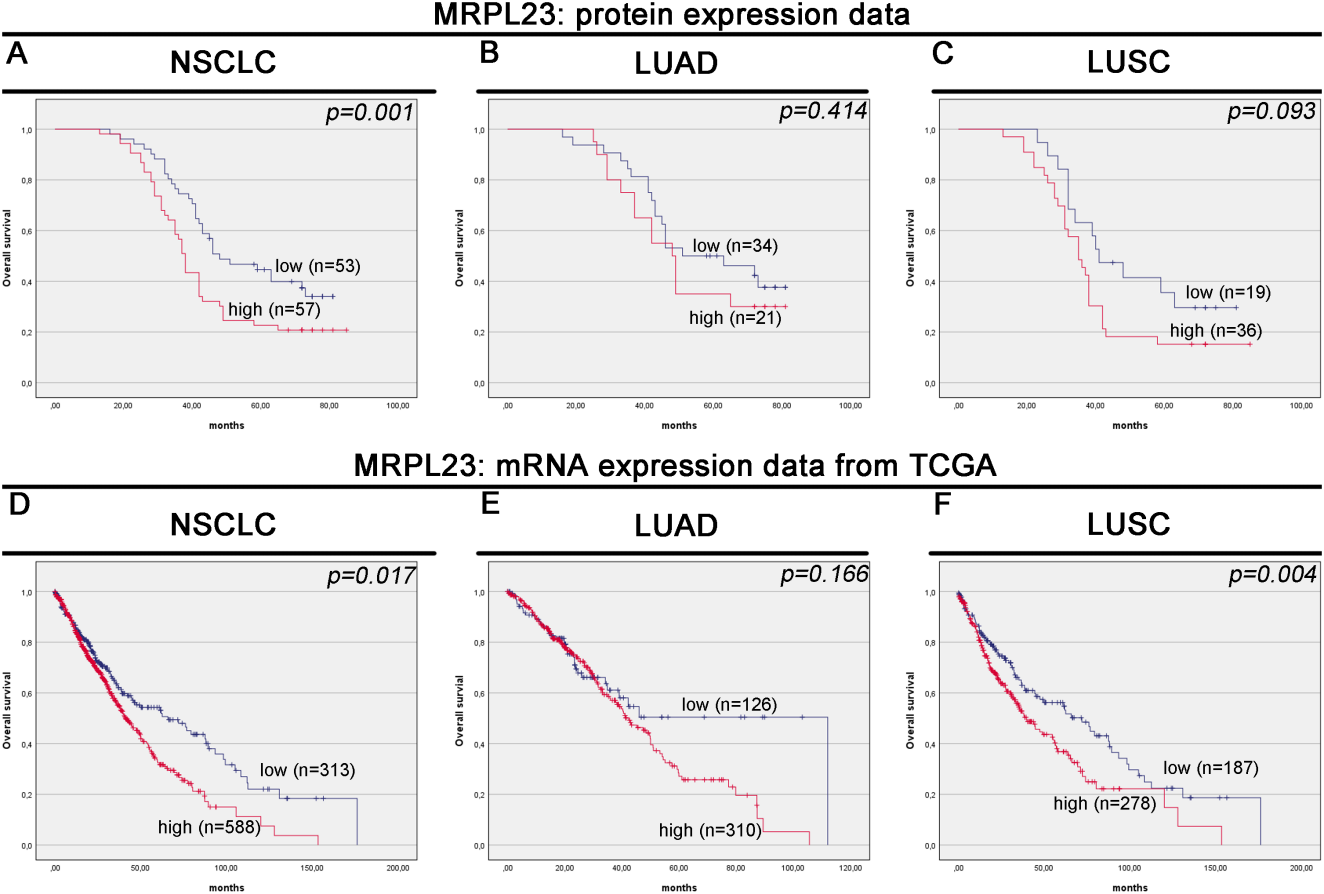
Kaplan-Meier survival curves for MRPL23 protein and mRNA expression in non-small cell lung cancer (NSCLC) tissues. Kaplan-Meier survival analysis for MRPL23 protein expression in NSCLC **(A)**, LUAD (lung adenocarcinoma) **(B)**, and LUSC (lung squamous cell carcinoma) **(C)**. Kaplan-Meier survival analysis for MRPL23 mRNA expression in NSCLC **(D)**, LUAD **(E)**, and LUSC **(F)**.

MRPL23 immunoreactivity was observed only in cancer cells (**Figure 1**), with normal lung tissue showing only negative or very weak cytoplasmic staining (panels A-B). Inter-observer concordance for MRPL23 IHC scoring was high (κ = 0.87; p < 0.001), confirming the reproducibility of the assessment. In tumor samples, panels C-D (LUAD) and panels E-F (LUSC) illustrate low MRPL23 protein expression, defined by weak cytoplasmic staining (IRS <8) in a limited proportion of tumor cells. In contrast, panels G-H (LUAD) and I-J (LUSC) demonstrate strong staining intensity and widespread cytoplasmic positivity (IRS ≥8), corresponding to high MRPL23 expression. Among 110 NSCLC cases, 57 (51.82%) showed high expression, while 53 (48.18%) had low expression. When comparing tumor tissue to normal lung, MRPL23 levels were significantly higher in NSCLC (p < 0.0001, **Figure 2A**). Expression differed significantly across histological subtypes, with LUSC displaying higher MRPL23 levels than LUAD (**Figure 2B**, **Table 1**). Kaplan-Meier analysis showed that high MRPL23 expression was associated with significantly shorter overall survival in the entire NSCLC cohort (median OS 34 vs 48 months; p = 0.001, **Figure 3A**). In LUAD (**Figure 3B**), survival did not differ significantly between high and low expression groups (p=0.414). In LUSC (**Figure 3C**), patients with high expression showed a trent toward poorer survival, though not reaching statistical significance (p=0.093). In univariate Cox analysis for the entire NSCLC cohort, high MRPL23 expression was significantly associated with worse prognosis (HR 1.73, 95% CI 1.09-2.74, p = 0.02; **Table 2**). This effect persisted in multivariate analysis after adjusting for age and clinical stage (HR 1.62, 95% CI 1.01-2.58, p = 0.04, **Table 2**).

**Table 1:** MRPL23 protein expression and its relationship with clinicopathological features of non-small cell lung cancer patients. .

Variables	Number (%)	MRPL23 Low	MRPL23 High	P value
		n=53	n =57	
Histological type				
Squamous cell carcinoma	55 (50.00)	19 (34.55)	36 (65.45)	0.01
Adenocarcinoma	55 (50.00)	34 (61.82)	21 (38.18)	
Gender				
Females	35 (31.82)	19 (54.29)	16 (45.71)	0.42
Males	75 (68.18)	34 (45.33)	41 (54.67)	
Age				
≤56	52 (47.27)	23 (44.23)	29 (55.77)	0.45
>56	58 (52.73)	30 (51.72)	28 (48.28)	
Grade				
Gx	5 (4.55)			
G1	3 (2.73)	1 (33.33)	2 (66.67)	0.26
G2	46 (41.82)	26 (56.52)	20 (43.48)	
G3	56 (50.91)	23 (41.07)	33 (58.93)	
pT status				
T1	3 (2.73)	0 (0.00)	3 (100.00)	0.17
T2	90 (81.82)	43 (47.78)	47 (52.22)	
T3	17 (15.45)	10 (58.82)	7 (41.18)	
pN status				
N0	65 (59.09)	35(53.85)	30 (46.15)	0.05
N1	40 (36.36)	18 (45.00)	22 (55.00)	
N2	5 (4.55)	0 (0.00)	5 (100.00)	
Stage				
I	51 (46.36)	27 (52.94)	24 (47.06)	0.27
II	45 (40.91)	22 (48.89)	23 (51.11)	
III	14 (12.73)	4 (38.57)	10 (71.43)	

**Table 2:** MRPL23 protein expression. Univariate and multivariate analyses of prognostic factors using the Cox proportional hazard model.

	Univariate analysis				Multivariate analysis			
Variable	HR	95% CI		P-value	HR	95% CI		P value
MRPL23	1.73	1.09	2.74	**0.02**	1.62	1.01	2.58	**0.04**
Gender	1.02	0.63	1.66	0.93	–	–	–	–
Age	2.00	1.24	3.23	**<0.01**	1.04	1.01	1.07	0.31
Grade	1.46	0.94	2.25	0.09	–	–	–	**-**
Stage	3.62	2.18	6.02	**<0.01**	3.38	1.9	5.58	**<0.01**

Bold values indicate statistically significant results (p < 0.05)

### MRPL23 mRNA expression and its association with clinical features and patient survival in the TCGA cohort

Out of 901 NSCLC samples in the TCGA dataset, 588 (65.26%) exhibited high and 313 (34.74%) low MRPL23 expression. mRNA levels were significantly higher in tumors tissues compared with normal lung (p < 0.0001, **Figure 2C**) and differed between histological subtypes, with LUSC showing higher expression than LUAD (**Figure 2D**, **Table 3**). After FDR adjustment (NSCLC vs normal; LUAD vs LUSC, m = 2), both results remained statistically significant (p < 0.05). Among clinicopathological parameters (age, sex pT, and pN; m = 5), a nominal association was noted with pT status (p = 0.04), but none remained significant after FDR adjustment. High MRPL23 mRNA expression correlated with shorter overall survival in the whole NSCLC cohort (median OS 41 vs 66 months; p = 0.017; **Figure 3D**) and remained significant in the LUSC subtype (p = 0.004; **Figure 3F**), while no difference was observed in LUAD (p = 0.166; **Figure 3E**). After FDR correction (m–= 3; NSCLC, LUAD, LUSC), the associations for the overall NSCLC cohort and for LUSC remained significant (q < 0.05). In univariate Cox analysis, high MRPL23 expression was associated with poorer overall survival (HR 1.46, 95% CI 1.16-1.83, p <0.01; **Table 4**) and remained an independent prognostic factor for shorter OS in NSCLC in multivariate model adjusted for pT and pN (HR 1.46, 95% CI 1.17-1.83, p <0.01; **Table 4**). A minor deviation from proportionality was observed for MRPL23 (p = 0.032). However, the Schoenfeld residual plot did not demonstrate a systematic time-dependent trend, and the deviations occurred primarily at the extremes of follow-up where event numbers were low. The global test did not indicate a violation of the proportional hazards assumption (p = 0.11), supporting the appropriateness of the Cox model (**Supplementary Figure 1**).

**Table 3:** MRPL23 mRNA expression and its relationship with clinicopathological features of non-small cell lung cancer patients in the TCGA cohort.

Variables	Number (%)	MRPL23 Low	MRPL23 High	P value
		n=313	n=588	
Histological type				
Squamous cell carcinoma	465 (51.61)	187 (40.22)	278 (59.78)	0.0005
Adenocarcinoma	436 (48.39)	126 (28.90)	310 (71.10)	
Age (years)				
<70	522 (57.94)	174 (33.33)	348 (66.67)	0.32
>70	379 (42.06)	139 (36.68)	240 (63.32)	
Gender				
Male	548 (60.82)	202 (36.86)	346 (63.32)	0.10
Female	353 (39.18)	111 (31.44)	242 (68.56)	
pT status				
1	257 (28.52)	75 (29.18)	182 (70.82)	0.04
2	504 (55.94)	183 (36.31)	321 (63.69)	
3	104 (11.54)	45 (43.27)	59 (56.73)	
4	36 (4.00)	10 (27.78)	26 (72.22)	
pN status				
N0	581 (64.48)	207 (35.63)	374 (64.37)	0.38
N1	207 (22.97)	75 (36.23)	132 (63.77)	
N2	106 (11.76)	29 (27.36)	77 (72.64)	
N3	7 (0.78)	2 (28.57)	5 (71.43)	
Stage				
I	460 (51.05)	160 (34.78)	300 (65.22)	0.90
II	110 (12.21)	35 (31.82)	75 (68.18)	
III	304 (33.74)	109 (35.86)	195 (64.14)	
IV	27 (3.00)	9 (33.33)	18 (66.67)	

**Table 4:** MRPL23 mRNA expression. Univariate and multivariate analyses of prognostic factors using the Cox proportional hazard model.

	Univariate analysis				Multivariate analysis			
Variable	HR	95% CI		P-value	HR	95% CI		P value
MRPL23	1.46	1.16	1.83	**<0.01**	1.46	1.17	1.83	**<0.01**
Gender	0.88	0.71	1.10	0.26	–	–	–	–
Age	1.01	1.00	1.02	0.20	–	–	–	–
pT status	1.97	1.53	2.53	**<0.01**	1.88	1.46	2.42	**<0.01**
N status	1.60	1.30	1.97	**<0.01**	1.51	1.23	1.86	**<0.01**

Bold values indicate statistically significant results (p < 0.05)

### MRPL23 protein and mRNA expression in cell lines

**Figure 4:**
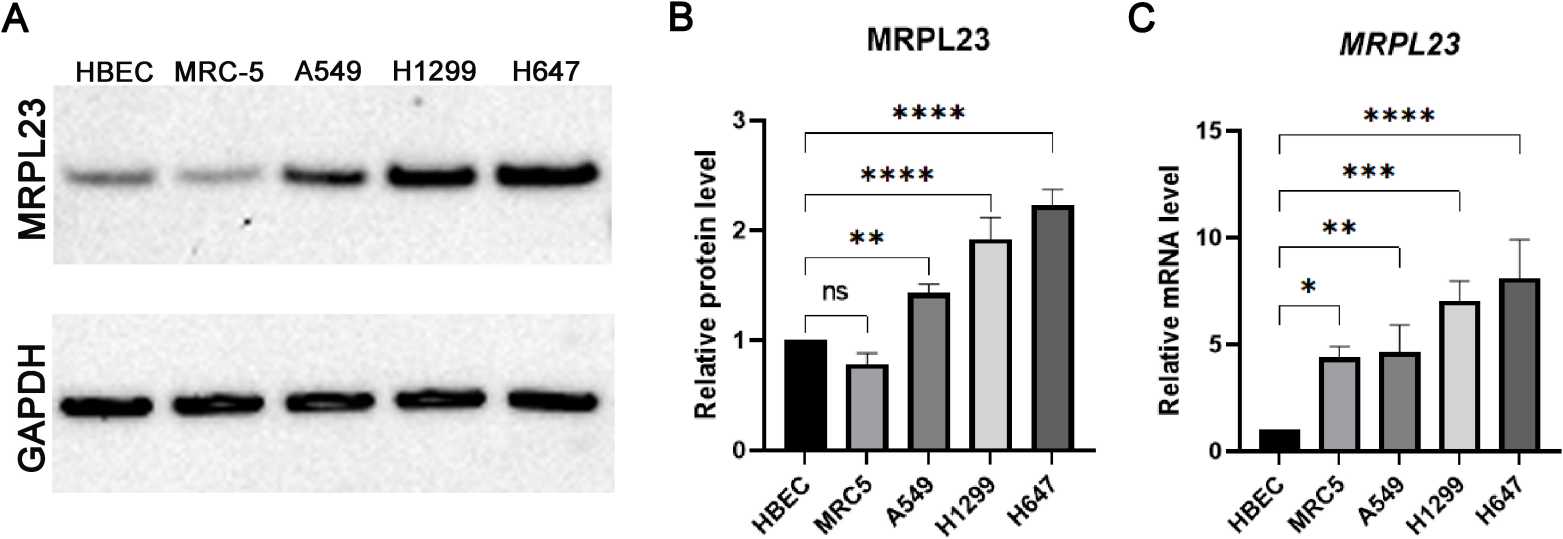
MRPL23 expression in normal and non-small cell lung cancer (NSCLC) cell lines. Western blot analysis of MRPL23 protein levels in normal bronchial epithelial cells (HBEC), normal lung fibroblasts (MRC-5), and non-small cell lung cancer cell lines (A549, H1299, H647), with GAPDH used as a loading control **(A)** Positive and negative controls were included in the experiment, and any cropping of the blot has been performed to enhance clarity while preserving important bands. The original, uncropped images of the blot, with visible gel edges, are provided in **Supplementary Figures S1 and S2**. Quantification of MRPL23 protein expression relative to GAPDH, based on Western blot data **(B)**. Relative mRNA expression of MRPL23 in the same cell lines, determined by qPCR **(C)**. ns: not significant, *p < 0.05, **p < 0.01, ***p < 0.001, ****p < 0.0001.

Western blotting confirmed markedly elevated MRPL23 protein levels in A549, H1299, H647 NSCLC cell lines compared with HBEC and MRC-5 non-malignant controls (**Figures 4A, B**). Among malignant lines, the highest expression was observed in the H647 cell line, followed by H1299 and A549, whereas HBEC and MRC-5 displayed much lower MRPL23 levels. GAPDH served as a loading control to verify equal protein input. Quantitative analysis demonstrated significantly increased MRPL23 protein levels in all cancer cell lines relative to HBEC (A549 p < 0.01; H1299 p < 0.0001; H647 p < 0.0001), while the difference between MRC-5 and HBEC was not significant. *Post-hoc* testing was performed using Dunnett’s correction for multiple comparisons versus the HBEC reference group (α = 0.05). Consistent with the protein data, qPCR analysis showed a parallel pattern at the mRNA level (**Figure 4C**). MRPL23 transcript levels were significantly up-regulated in all NSCLC cell lines compared with HBEC (A549 p < 0.01; H1299 p < 0.001; H647 p < 0.0001), with MRC-5 also showing a moderate but significant increase (p < 0.05).

## Discussion

Diverse roles of MRPL23 across different cancer types underscore its potential as a therapeutic target and prognostic biomarker. Recent scientific reports have identified MRPL23 expression as a promising prognostic biomarker, particularly in adenoid cystic carcinoma and osteosarcoma (17, 18). Given this background, our research seeks to bridge a significant gap in the understanding of MRPL23’s role in NSCLC. According to our best knowledge, our study is the first to investigate the prognostic significance of MRPL23 protein in NSCLC. Here, we employed a comprehensive suite of laboratory techniques, including tissue microarrays, IHC staining, Western blot, and PCR, to assess the expression of MRPL23 mRNA and protein. Our analysis revealed significant differences in MRPL23 expression levels, both within our cohort and in silico analyses, comparing tumor samples to control tissues. The results of our study indicated higher expression of MRPL23 in tumor tissues compared to non-tumor tissues. This phenomenon was evident in the LUSC subtype of lung cancer, suggesting its potential role in carcinogenesis. Although high MRPL23 expression significantly correlated with shorter overall survival in the entire NSCLC cohort, this association did not reach statistical significance in analyses restricted to LUAD or LUSC subgroups. This is likely due to the smaller sample sizes of these subgroups, and therefore limited statistical power to detect significant differences despite observable trends. Notably, analysis of the TCGA LUSC cohort confirmed a statistically significant association between high MRPL23 expression and poorer overall survival (**Figure 3F**). Studies on larger and independent cohorts are warranted to validate these findings.

While we observed no association between MRPL23 status and clinicopathological features of NSCLC, survival analysis revealed that heightened MRPL23 expression was associated with diminished overall survival, a relationship further supported by multivariate Cox modelling, confirming that MRPL23 functions as an independent prognostic factor. Although the corresponding hazard ratios represent moderate effect sizes, their magnitude is comparable to other metabolic and mitochondrial biomarkers investigated in NSCLC (10). In the TMA cohort, high MRPL23 expression translated into a 62% increase in mortality risk after adjustment for age and clinical stage (HR 1.62, 95% CI 1.01-2.58). In the larger TCGA cohort, the effect was slightly attenuated (HR 1.46, 95%CI 1.17-1.83), but its persistence in multivariate modeling supports its robustness. Such hazard ratios are also consistent with those reported for other mitochondrial and metabolic markers, including MRPL13 and protein implicated in OXPHOS dysregulation and oxidative-stress signaling, suggesting that MRPL23 falls within the expected effect spectrum for this class of biomarkers (8). In practical terms, hazard ratios of this magnitude may still carry relevance for clinical decision-making, particularly in early-stage NSCLC, where traditional clinicopathological parameters often fail to fully capture biological heterogeneity and additional prognostic discrimination is needed.

Our observations align with expression levels in the TCGA cohort, further confirming their reliability. The outcomes suggest that MRPL23 may not only serve as a significant prognostic factor in lung cancer patients, but also has the potential to contribute to advancements in personalized therapy and enhance the quality of patient care. From a clinical perspective, MRPL23 should not be viewed as a replacement for established prognostic markers but rather as a complementary indicator that may help identify patients who, despite favorable baseline characteristics, harbor a more aggressive disease biology and could benefit from closer surveillance or more intensive adjuvant management. Although therapeutic implications remain speculative, the link between MRPL23 and mitochondrial/oxidative stress pathways suggests that it may become relevant in future strategies targeting mitochondrial vulnerability in NSCLC.

Zhong X. et al. demonstrated that MRPL13 expression was significantly upregulated in lung adenocarcinoma compared to control tissues, which was associated with poor prognosis. The correlation analysis indicated that MRPL13 expression may promote tumor progression by suppressing the expression of protective genes, such as NIFX and CBX7. Downregulation of these protective genes contributes to enhanced tumor growth, increased cancer cell survival, and metastasis development, ultimately leading to increased tumor malignancy. Lower MRPL13 expression also correlated with a more favorable anti-tumor microenvironment. Notably, the repression of MRPL13 expression was associated with reduced cancer cell viability (7). Similarly, Jing C. et al. noted that reduced MRPL13 expression inhibited tumor cell proliferation (19). Zeng Y. et al. focused on the role of another member of this family, MRPL15, observing that higher MRPL15 expression was correlated with lower tumor infiltration and mutations in the TP53 gene, a critical tumor suppressor. Moreover, elevated MRPL15 expression was associated with poorer NSCLC clinical outcomes, including shorter OS, progression-free survival (PFS), relapse-free survival (RFS), and disease-free survival (DFS). Additionally, MRPL15 expression differed between genders, clinical stages, and lymph node status groups (8).

Unfortunately, while other MRPL family proteins have been shown to play a significant role in NSCLC carcinogenesis, the mechanistic role of MRPL23 in lung cancer has not yet been described. However, in our previous study, we assessed the role of MRPL23 in clear cell renal cell carcinoma (ccRCC), where significantly lower protein expression levels were observed in patients with ccRCC. In contrast, TCGA data analysis revealed higher mRNA expression in tumor samples, suggesting that carcinogenesis is not solely influenced by the isolated expression of a single protein, but also by its co-expression and interactions with the immune system and the tumor microenvironment (12).

While biological research is still expanding our understanding of MRPL23’s role in oncogenesis, MRPL23 Antisense RNA 1 (MRPL23-AS1) has been extensively studied in this context. While both molecules are located on chromosome 11p15.5, they serve distinct biological functions: MRPL23 is involved in protein synthesis and cellular metabolism, whereas MRPL23-AS1 functions as a long non-coding RNA (lncRNA). Hence, they are likely functionally interdependent in regulating oncogenic processes (20). Studies on adenoid cystic carcinoma (ACC) have shown that increased MRPL23-AS1 expression was associated with advanced clinical stage, lung metastases, and lower overall survival rates. The study also revealed a positive association between the expression levels of MRPL23-AS1 and the EREG gene, a known determinant of lung metastasis. This correlation remained consistently observable across both salivary adenoid cystic carcinoma (SACC) cell lines and tissue specimens. This effect appears linked to the promotion of epithelial-mesenchymal transition (EMT) by MRPL23-AS1 and its interactions with EZH2 and H3K27me3 in the E-cadherin promoter, thereby promoting metastasis ​ (18). The ceRNA hypothesis, introduced by Salmena et al., describes how mRNA and lncRNA can communicate via microRNA response elements, competing for microRNA binding and influencing carcinogenesis.

In lung cancer, oncogenic lncRNAs are typically upregulated and bind directly to tumor-suppressive miRNA, promoting cancer cell growth and development ​ (21). MRPL23-AS1 has also been shown to participate in the ceRNA network, as demonstrated by X Tan et al. in colorectal cancer, where it was recognized as an independent prognostic factor (22). Li et al. further reported that MRPL23-AS1 promotes salivary adenoid cystic carcinoma cell resistance to anoikis, a process crucial for metastasis. The study unveiled that MRPL23-AS1 exerts epigenetic control over the expression of the p19INK4D gene, associated with resistance to anoikis. These findings highlight MRPL23-AS1’s role in tumor progression and suggest MRPL23 as a potential therapeutic target or biomarker (23). It’s indeed intriguing how distinct molecules, such as MRPL23-AS1 and MRPL13, exert similar effects on lung cancer cells invasion and migration, impacting patients prognosis (7). However, studies such as that by Yong et al. contradict the hypothesis that elevated lncRNA expression universally corresponds to poorer patient prognosis and disease advancement. In gastric cancer, decreased lncRNA HMlincRNA717 expression correlated with distant metastasis, venous invasion, and nerve invasion (24). Similarly, low expression of the lncRNA ZNF385D−AS2 was an independent prognostic factor of shorter overall survival in hepatocellular carcinoma. Interestingly, Zhang et al. also suggested that ZNF385D−AS2 is involved in the RNA communication network, which may have significant implications for cancer development (25). Despite similar mechanisms involved in tumorigenesis and metastasis, lncRNA expression levels appear to impact tumor development in a context-dependent manner, likely reflecting differences in tumor suppression profiles, study cohort characteristics, or immune response *in vitro*.

Although direct mechanistic studies of MRPL23 in NSCLC are lacking, our findings, combined with existing evidence from other cancers and mitochondrial biology, suggest plausible pathways by which MRPL23 overexpression could contribute to lung cancer progression. However, we acknowledge that our study does not include functional assays such as MRPL23 knockdown or overexpression, nor pathway interrogation to experimentally validate the proposed mechanisms involving metabolic reprogramming or ROS modulation. Nevertheless, previous studies on related MRPL family members, such as MRPL13 and MRPL15, have demonstrated their ability to regulate cancer cell proliferation, apoptosis resistance, and metabolic adaptation. Based on these reports, it is reasonable to hypothesize that MRPL23 may play a similar functional role in NSCLC. Future *in vitro* and *in vivo* studies are therefore warranted to determine whether MRPL23 directly influences key oncogenic processes and to establish whether it acts as an active driver of tumor progression rather than a passive biomarker. Since MRPL23 plays a crucial role in the translation of mitochondrial DNA–encoded proteins that are essential for oxidative phosphorylation, its dysregulation may impair this process and promote a shift toward anaerobic glycolysis (Warburg effect), supporting tumor cell proliferation and survival under hypoxic conditions (10–14). Moreover, mitochondrial dysfunction associated with aberrant MRPL23 levels could disrupt reactive oxygen species (ROS) balance, thereby modulating signaling pathways involved in apoptosis resistance, proliferation, and genomic stability (15, 16). Elevated MRPL23 expression, as observed in our study and reported in other cancers, may enhance tumor cell survival by promoting energy production, reducing susceptibility to apoptosis, and fostering a microenvironment that facilitates disease progression (12). These hypotheses require functional validation in NSCLC but provide a framework for understanding how MRPL23 could mechanistically link to poor prognosis in this malignancy.

Gaining a deeper comprehension of the interplay between MRPL family proteins and carcinogenesis bears significant clinical relevance. Alterations in the expression of MRPL23, MRPL23-AS1, and related MRPLs impact tumorigenesis, metastasis, and clinical-pathological features across cancers. Comparable mechanisms may operate in NSCLC, but comprehensive investigations are lacking. Future research should focus on *in vitro* and *in vivo* models to clarify MRPL23’s role in NSCLC progression, including its effects on proliferation, migration, invasion, signaling pathways, and response to therapy. Additionally, studies on differential MRPL23 expression in LUAD, its subtype-specific roles, and interactions with other biomarkers will help define its potential in personalized medicine.

It should be noted that although our TMA cohort provided sufficient statistical power for the overall NSCLC analysis, the subgroup analyses for LUAD and LUSC were likely underpowered. The lack of statistical significance in survival comparisons within these subgroups, despite observable trends, should therefore be interpreted with caution. Larger histology-specific cohorts will be required to fully validate the prognostic relevance of MRPL23 across NSCLC subtypes.

Nonetheless, our study is not devoid of limitations. We acknowledge the relatively modest size of our sample cohort, underscoring the imperative for broader investigations encompassing larger and ethnically diverse populations. Furthermore, there is a risk that the results of cell culture studies may not fully correspond to those obtained *in vivo* due to specific mechanisms and environmental conditions. Moreover, the lack of broader data on factors such as patients’ performance status and the presence of driver mutations represents a limitation of our study. Despite these limitations, we have endeavored to conduct the study with due diligence. The results highlight the importance of MRPL23 as a potential biomarker and therapeutic target, thus paving the way for future research that could ultimately lead to the identification of new therapeutic strategies.

## Materials and methods

### Tissue microarrays

Tissue Microarray (TMA) section was acquired from a commercial supplier (US Biomax, Rockville, MD, USA, LUC1201MSur). The TMA slide included specimens from 120 patients diagnosed with NSCLC carcinoma (55 cases of squamous cell carcinoma and 55 cases of LUAD), along with 10 cases of normal lung tissue. Clinicopathological features included in the study were: histological type, gender, age, grade, pT status, pN status, and stage. The OS data reflect survival measured from the time of diagnosis to death. The detailed characterization of the study group is shown in **Supplementary Table S1**.

### Cell culture and reagents

The following human NSCLC cell lines (A549, H1299, H647), normal human bronchial epithelial cell line (HBEC), and normal human diploid fibroblast cells (MRC-5) were purchased from the American Type Culture Collection (ATCC, Manassas, VA, USA) and cultured in monolayers at 37 °C in a humidified CO2 incubator (5% CO2). A549 cell line was cultivated in Dulbecco’s Modified Eagle’s Medium (DMEM, Corning, catalog no. 10-013-CV) supplemented with 10% fetal bovine serum (FBS, Sigma-Aldrich, catalog no. F9665) and 1% Penicillin Streptomycin Solution (P/S, Corning, catalog no. 30-002-CI). Both H647 and H1299 cell lines were cultured in Roswell Park Memorial Institute 1640 Medium (RPMI 1640, Corning, catalog no. 10-040-CV) supplemented with 10% FBS and 1% P/S. The HBEC cell line was cultured in Bronchial/Tracheal Epithelial Cell Growth Medium: All In One ready to use (HBEpC/HTEpC Growth Medium, Cell Applications, INC., catalog no. 511-500) supplemented with Retinoic Acid 0.1 µg/ml (RA, Cell Applications, INC., catalog no. 511-RA). MRC-5 cells were maintained in Eagle’s Minimum Essential Medium (EMEM, Corning, catalog no. 10-009-CV) supplemented with 10% FBS and 1% P/S. All cell lines used in this study were authenticated and tested for mycoplasma contamination.

### In silico analysis

The mRNA levels of MRPL23 were obtained from the TCGA and GTEx cohorts (26). Gene expression, survival, and clinicopathological data for a cohort of 901 NSCLC patients were retrieved from the UCSC Xena Browser (http://xena.ucsc.edu/, accessed July 15, 2024). TCGA and GTEx expression data were used in the Toil-harmonized version provided by UCSC Xena, ensuring a consistent preprocessing pipeline across datasets. RNA-seq expression values were analysed as DESeq2-normalized counts and log2-transformed prior to downstream statistical analyses. Detailed characteristics of the study group are presented in **Supplementary Table S2**. The cut-off points for MRPL23 mRNA expression were determined using the Evaluate Cutpoints software, which stratified the samples into low- and high-expression groups (27). The threshold for MRPL23 expression was established at < 10.04 for low expression and ≥ 10.04 for high expression.

### Immunohistochemical staining and analysis

The TMA slide underwent immunohistochemical staining for the evaluation of MRPL23 protein expression. Rabbit polyclonal anti-MRPL23 antibody (Sigma-Aldrich, catalog no. HPA050406) was used at a dilution of 1:100 with an incubation time of 32 minutes. Staining optimization was performed according to the manufacturer’s instructions. The staining procedure was carried out using the BenchMark ULTRA system (Roche Diagnostics/Ventana Medical Systems, Tucson, AZ, USA), and visualization was achieved using the OptiView DAB IHC Detection Kit (Roche Diagnostics/Ventana Medical Systems, Tucson, AZ, USA). The TMA slide was digitized using a slide imaging scanner, Roche Ventana DP 200 (Roche Diagnostics/Ventana Medical Systems, Tucson, AZ, USA). The semi-quantitative assessment of protein expression levels was conducted by a pathomorphologist and a scientist. One core per patient was evaluated for MRPL23 expression. MRPL23 immunohistochemical staining was present in the cytoplasm. The evaluation utilized a modified Remmele-Stegner index scale (IRS), with a final score ranging from 0 to 12, calculated by multiplying the percentage of positively stained cells or areas (ranging from 0 to 4) by the intensity of staining (ranging from 0 to 3). The cut-off points were established using the Evaluate Cutpoints software, which stratified the data into low and high expression groups. The threshold for MRPL23 protein expression was set at < 8 for low expression and ≥ 8 for high expression.

### Western blot analysis

Cell lysates were prepared from three independent cultures of each cell line using RIPA Lysis and Extraction Buffer (Thermo Fisher Scientific, catalog no. 89900), supplemented with protease inhibitors (Halt™ Protease Inhibitor Cocktail, Thermo Fisher Scientific, catalog no. 87786). Protein concentrations were measured using the Qubit™ Protein Assay Kit (Thermo Fisher Scientific, catalog no. Q33212), following the manufacturer’s instructions. Protein samples were then prepared for electrophoresis by adding NuPAGE™ LDS Sample Buffer (Thermo Fisher Scientific, catalog no. NP0007) and NuPAGE™ Sample Reducing Agent (Thermo Fisher Scientific, catalog no. NP0009). Samples were subjected to electrophoresis on NuPAGE™ Bis-Tris gels with a gradient of 4-12% (Thermo Fisher Scientific, catalog no. NP032B) until fully separated by molecular weight, then transferred to a nitrocellulose membrane using the iBlot™ Dry Blotting System (Thermo Fisher Scientific, catalog no. IB21001). Before incubation with antibody, the membrane was blocked with 5% non-fat milk in TBST to prevent non-specific binding. Rabbit anti-MRPL23 polyclonal antibody (Proteintech, catalog no. 11706-1-AP) was used at a dilution of 1:2000. The membranes were incubated overnight at 4 °C with this antibody, followed by incubation with Goat anti-Rabbit IgG (H+L) Secondary Antibody, HRP conjugate (Thermo Fisher Scientific, catalog no. 31460) at a dilution of 1:5000 for 1 hour. After several washes with TBST buffer, immunoreactive bands were visualized using the SuperSignal™ West Pico PLUS Chemiluminescent Substrate (Thermo Fisher Scientific, catalog no. 34580) and detected using the iBright™ Imaging System (Thermo Fisher Scientific). GAPDH Loading Control Monoclonal Antibody (GA1R, Thermo Fisher Scientific, catalog no. MA515738HRP) was used as a loading control. Quantitative analysis of the bands was performed using ImageJ software.

### Quantitative-RT-PCR

Total RNA was extracted from cell cultures using the High Pure RNA Isolation Kit (Roche Diagnostics, catalog no. 11828665001). Complementary DNA synthesis was performed with the Transcriptor First Strand cDNA Synthesis Kit (Roche Diagnostics, catalog no. 04379012001). For quantitative real-time PCR (qRT-PCR), the LightCycler 480 SYBR Green I Master (Roche Diagnostics, catalog no. 04707516001) was used on the cobas z 480 analyzer. The relative expression of the MRPL23 gene was calculated using the ΔΔCt method (delta-delta Ct), with normalization to the reference gene GAPDH. The primer sequences for the MRPL23 gene were ordered from Genomed S.A. The sequences were as follows: MRPL23-F (forward primer) 5’-GACAAGGGTGGACCTCAGGAAT-3’, MRPL23-R (reverse primer) 5’-CGGCTTCTTGATCCTCACGTTTC-3’. The primer sequences for the reference gene GAPDH were: GAPDH-F (forward primer) 5’-GAAGGTGAAGGTCGGAGTC-3’, GAPDH-R (reverse primer) 5’-GAAGATGGTGATGGGATTTC-3’.

### Statistical analyses

The normality of continuous data was assessed using the Shapiro–Wilk test. Comparative analyses of continuous variables were performed with the Mann–Whitney U test, while categorical variables were evaluated using the Chi-squared or Fisher’s exact tests, as appropriate. For survival endpoints, the Holm–Bonferroni method was applied. To account for multiple comparisons within each family of exploratory analyses, p-values were adjusted using the Benjamini–Hochberg false discovery rate correction (FDR, q = 0.05; **Supplementary Tables 3–8**). Survival curves were generated using the Kaplan–Meier method and compared using the Mantel–Cox log-rank test. Univariate and multivariate Cox proportional hazards models were used to estimate hazard ratios (HR) with 95% confidence intervals (CI). The proportional hazards assumption was evaluated using the Cox proportional hazards test and Schoenfeld residuals. Because the Cox model jointly estimates covariate effects, no multiple-testing correction was applied to multivariable analyses (**Supplementary Tables 9–10**). A significance level of p < 0.05 was considered statistically significant. The statistical analyses were executed using GraphPad Prism (v8.0; GraphPad Software, San Diego, CA, USA), Statistica v13.3 (StatSoft), and the SPSS software packages (v26.0, IBM Corporation, Armonk, NY, USA).

## Data Availability

The data presented in this study are deposited in the Zenodo repository, accession number 10.5281/zenodo.18324703.
