# Supplementary material for: Identification of candidate biomarker MRPL23 and its prognostic potential in non-small cell lung cancer with emphasis on the squamous cell carcinoma subtype
Source: Front Oncol. 2026 Feb 2;16:1663235. doi: 10.3389/fonc.2026.1663235 (PMC12907143; doi:10.3389/fonc.2026.1663235)

Supplementary Table S1. Detailed characteristics of the TMA study group.

| **Variables** | **Number (%)** |
| --- | --- |
|  |  |
|  |  |
| **Histological type** |  |
| **Squamous cell carcinoma** | 55 (50.00) |
| **Adenocarcinoma** | 55 (50.00) |
| **Gender** |  |
| **Females** | 35 (31.82) |
| **Males** | 75 (68.18) |
| **Age** |  |
| **≤56** | 52 (47.27) |
| **>56** | 58 (52.73) |
| **Grade** |  |
| **Gx** | 5 (4.55) |
| **G1** | 3 (2.73) |
| **G2** | 46 (41.82) |
| **G3** | 56 (50.91) |
| **pT status** |  |
| **T1** | 3 (2.73) |
| **T2** | 90 (81.82) |
| **T3** | 17 (15.45) |
| **pN status** |  |
| **N0** | 65 (59.09) |
| **N1** | 40 (36.36) |
| **N2** | 5 (4.55) |
| **Stage** |  |
| **I** | 51 (46.36) |
| **II** | 45 (40.91) |
| **III** | 14 (12.73) |

Supplementary Table S2. Detailed characteristics of the TCGA study group.

| **Variables** | **Number (%)** |
| --- | --- |
|  |  |
|  |  |
| **Age (years)** |  |
| **<70** | 522 (57.94) |
| **>70** | 379 (42.06) |
| **Gender** |  |
| **Male** | 548 (60.82) |
| **Female** | 353 (39.18) |
| **pT status** | |
| **1** | 257 (28.52) |
| **2** | 504 (55.94) |
| **3** | 104 (11.54) |
| **4** | 36 (4.00) |
| **pN status** |  |
| **N0** | 581 (64.48) |
| **N1** | 207 (22.97) |
| **N2** | 106 (11.76) |
| **N3** | 7 (0.78) |
| **Stage** |  |
| **I** | 460 (51.05) |
| **II** | 110 (12.21) |
| **III** | 304 (33.74) |
| **IV** | 27 (3.00) |

Commentary to Supplementary Table 3-7

Tests were grouped into logical families and corrected using the Benjamini–Hochberg false discovery rate (FDR). Adjusted p-values (q-values) and individual significance thresholds are reported for each family.

Supplementary Table 3. Expression-level analyses (protein, TMA cohort)

| **Cohort** | **Raw p** | **Rank** | **BH threshold (q=0.05)** | **Adjusted p** | **Significant (q<0.05)** |
| --- | --- | --- | --- | --- | --- |
| NSCLC vs normal | <0.0001 | 1 | 0.025 | <0.0002 | Yes |
| LUSC vs LUAD | 0.01 | 2 | 0.05 | 0.01 | Yes |

Family size: m = 2 (NSCLC vs normal; LUSC vs LUAD)

Supplementary Table 4. Expression-level analyses (mRNA, TCGA cohort)

| **Cohort** | **Raw p** | **Rank** | **BH threshold (q=0.05)** | **Adjusted p** | **Significant (q<0.05)** |
| --- | --- | --- | --- | --- | --- |
| NSCLC vs normal | <0.0001 | 1 | 0.025 | <0.0002 | Yes |
| LUSC vs LUAD | <0.0001 | 2 | 0.05 | <0.0002 | Yes |

Family size: m = 2 (NSCLC vs normal; LUSC vs LUAD)

|  |
| --- |

Supplementary Table 5. Clinicopathological correlations (TMA cohort)

| **Variable** | **Raw p** | **Rank** | **BH threshold (q=0.05)** | **Adjusted p** | **Significant (q<0.05)** | **Significant (q<0.10)** |
| --- | --- | --- | --- | --- | --- | --- |
| Histological type | 0.0005 | 1 | 0.00833 | 0.003 | **Yes** | **Yes** |
| pT status | 0.04 | 2 | 0.01667 | 0.12 | No | No |
| Gender | 0.10 | 3 | 0.025 | 0.2 | No | No |
| Age | 0.32 | 4 | 0.03333 | 0.456 | No | No |
| pN status | 0.38 | 5 | 0.04167 | 0.456 | No | No |
| Stage | 0.90 | 6 | 0.05 | 0.90 | No | No |

Family size: m = 7 (histology, gender, age, grade, pT, pN, stage)

Supplementary Table 6. Clinicopathological correlations (TCGA cohort)

| **Variable** | **Raw p** | **Rank** | **BH threshold (q=0.05)** | **Adjusted p** | **Significant (q<0.05)** | **Significant (q<0.10)** |
| --- | --- | --- | --- | --- | --- | --- |
| Histological type | 0.01 | 1 | 0.0071 | 0.07 | No | **Yes** |
| pN status | 0.05 | 2 | 0.0143 | 0.175 | No | No |
| pT status | 0.17 | 3 | 0.0214 | 0.397 | No | No |
| Grade | 0.26 | 4 | 0.0286 | 0.455 | No | No |
| Stage | 0.27 | 5 | 0.0357 | 0.378 | No | No |
| Gender | 0.42 | 6 | 0.0429 | 0.49 | No | No |
| Age | 0.45 | 7 | 0.05 | 0.45 | No | No |

Family size: m = 7 (histology, gender, age, grade, pT, pN, stage)

Supplementary Table 7. Survival analyses (protein, TMA cohort)

| **Cohort** | **Raw p** | **Rank** | **BH threshold (q=0.05)** | **Adjusted p** | **Significant (q<0.05)** |
| --- | --- | --- | --- | --- | --- |
| NSCLC | 0.001 | 1 | 0.0167 | 0.003 | Yes |
| LUSC | 0.093 | 2 | 0.0333 | 0.14 | No |
| LUAD | 0.414 | 3 | 0.05 | 0.414 | No |

Family size: m = 3 (NSCLC, LUAD, LUSC KM comparisons)

Supplementary Table 8. Survival analyses (mRNA, TCGA cohort)

| **Cohort** | **Raw p** | **Rank** | **BH threshold (q=0.05)** | **Adjusted p** | **Significant (q<0.05)** |
| --- | --- | --- | --- | --- | --- |
| LUSC | 0.004 | 1 | 0.0167 | 0.012 | Yes |
| NSCLC | 0.017 | 2 | 0.0333 | 0.026 | Yes |
| LUAD | 0.166 | 3 | 0.05 | 0.166 | No |

Family size: m = 3 (NSCLC, LUAD, LUSC KM comparisons)

**Supplementary Table 9.** Proportional hazards test results for the TCGA multivariable Cox model

| **Variable** | **Chi-square** | **df** | **p-value** |
| --- | --- | --- | --- |
| **Lymph nodes N (N0 vs N+)** | 0.121 | 1 | 0.7278 |
| **MRPL23 (high vs low)** | 4.620 | 1 | **0.0316** |
| **Tumor stage (T1–T2 vs T3–T4)** | 1.126 | 1 | 0.2887 |
| **Global test** | 6.035 | 3 | 0.1099 |

**Supplementary Table 10.** Proportional hazards test results for the TMA multivariable Cox model

| **Variable** | **Chi-square** | **df** | **p-value** |
| --- | --- | --- | --- |
| **Stage (I vs. II-III)** | 0.105 | 1 | 0.7464 |
| **Age (<56 vs, ≥56)** | 3.479 | 1 | 0.0621 |
| **MRPL23 (Low vs. High)** | 2.678 | 1 | 0.1017 |
| **Global test** | 5.619 | 3 | 0.1317 |

Supplementary Figure 1. Shoenfeld rests for MRPL23 (TCGA cohort)

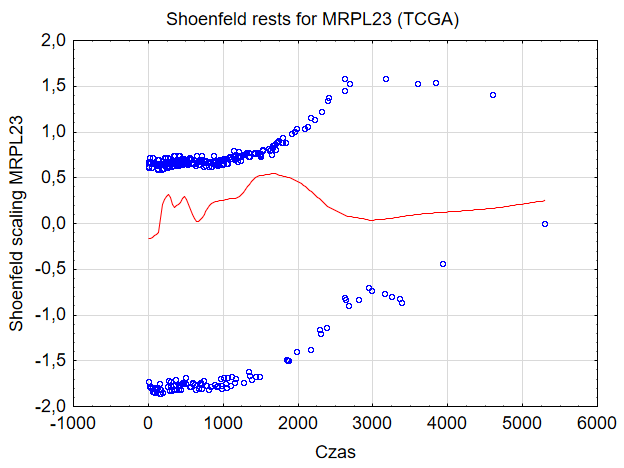

Supplement: Supplementary file 1 [file DataSheet1.docx]
